# Role of Aqueous Crude Leaf Extract of Senecio Biafrae Combined With Zinc on Testicular Function of Adult Male Sprague Dawley Rats 

**Published:** 2018-03

**Authors:** Sunday A Adelakun, Babatunde Ogunlade, Olusegun D Omotoso, Oyebowale O Oyewo

**Affiliations:** 1 Department of Human Anatomy, School of Health and Health Technology, Federal University of Technology, Akure, Nigeria; 2 Department of Anatomy, Kogi State University, Anyigba, Kogi State, Nigeria; 3 Department of Anatomy, Faculty of Basic Medical Sciences, Ladoke Akintola University of Technology, Ogbomoso, Nigeria

**Keywords:** Seneciobiafrae, Zinc, Lumen, Spermatozoa, Testosterone

## Abstract

**Objective:** To determine the role of aqueous crude leaf extract of *Seneciobiafrae *(*SB*) combined with Zinc (Zn) on Testicular function of Adult Male Sprague dawley Rats.

**Materials and methods:** Twenty-four adult males praguedawley Rats weighing 180-200g, aged 10-12 weeks, were randomized into four groups (A,B,C,D) of six rats each (n = 6) and were given 2mls of distilled water; 500 mg/kg of *SB *; 500 mg/kg of *SB* and 0.5 mg/kg Znsulfate; 500 mg/kg of *SB* and 1mg/kg of Zn. The administration was done daily via gastric gavage for 28 days. Parameterstested include: testicular histology, sperm parameters, haematological parameters and Testosterone assay.

**Results:** There was observed no significant (p > 0.05) increased in testis, epididymal, seminal vesicle, vas deference and prostate gland weight of animals across the group receiving the *Seneciobiafrae *extract combine with Zn. Testis and serum testosterone levels, sperm count, percentage of sperm viability and motility was higher in the rats administered a combination of *Seneciobiafrae *extract and Zn compared with controls (p < 0.05). Conversely the percentage of abnormal sperm morphology was decreased (p < 0.05). Histological analysis showed normal spermatogenesis, better association and high density of spermatogenic cells and lumen contains full mature spermatozoa.

**Conclusion:** Aqueous extract of *Seneciobiafrae *combined with Zn, potentially enhance testicular function including steroidogenesis and spermatogenesis in male rats.

## Introduction

*Seneciobiafrae *(local name worowo) belong to the group of vegetables that grow in large quantity as undercover in tree crop plantation, this leafy vegetable is also considered for its high medicinal value as the juice extracted from the leaves are wholly applied to fresh wounds or cuts as styptic in the rural community for man and animal use (1, 2). It is one of the green leafy vegetables consumed in Nigeria, Ghana, Benin, Sierra Leone, Cameroon and Gabon (3). It is consumed with pepper and onion, also eaten as streamed vegetables in combination with okro and fish (4). They are especially popular in south-western Nigeria. Nigeria is recognized worldwide for its vast fauna and flora biodiversity, which can be explored in several ways (i.e. culinary, medicinal, therapeutic, nutritional, etc.) for the benefit of mankind. A wide range of vegetable species from Nigeria’s flora have been used in folklore medicine for the treatment of several maladies both in the “old and new world” (5). Green leafy vegetables provide a source of vitamins, minerals and fiber for the local consumers. Due to their dietary importance, many scientific studies have been carried out on the potentials of these green leaves (6). Green leafy vegetables are medicinal and about 80% of the population in developing countries use medicinal plants and plant products in handling some there primary medical problems, due to their accessibility, availability and affordability (7, 8). In these countries, a variety of plants are claimed to have fertility regulating properties and a few have been tested for such effects (8, 9).

Zinc (Zn) is an essential trace element for living organisms. More than 300 enzymes rely on Zn for their functions. It also plays an important role in the DNA replication, transcription, and protein synthesis, influencing cell division and differentiation (10). It has been reported that Zn has a relationship with many enzymes in the body and can prevent cell damage through activation of the antioxidant system (11, 12, 13). Zinc is an essential component of the oxidant defense system and functions at many levels (14, 15). It was also revealed to inhibit generation of reactive oxygen species and enhance the activity of antioxidant pathways (16). Zn deficiency in the diet paves the way for cell damage in the rat testis (17). Furthermore, Zn deficiency increases lipid peroxidation in various rat tissues, whereas the Zn supplementation corrects the impairment (13, 18). These study focus on the effect of *Seneciobiafrae* extract in combination with zinc on the testicular function of adult spraguedawley rats.

## Materials and methods

***Collection of plant:*** Plant materials were collected from the Research Farm, Faculty of Agricultural Sciences, Ladoke Akintola University of Technology (LAUTECH) Ogbomoso, OyoState., Nigeria in Jun, 2016. Samples of *Seneciobiafrae* were identified and authenticated by Prof. A.J. Ogunkunle of the Department of Pure and Applied Biology.

***Preparation of plant extracts:*** The extraction of plant was done as explained by Muhammed *et al.,* (19) The air-dried leaves were weighed using Gallenkamp (FA2104A, England) electronic weighing balance and were milled with VTLC electrical Blender (model - Smart Leaf, India) to powdered form. Aqueous extract was prepared by soaking dry powder in 1:10 ratio in distilled water for 72 h with intermittent shaking. After 72 h of soaking the content was filtered through cheese cloth and What man filter paper (0.45 µm) (20, 21) at room temperature and filtrate was concentrated under reduced pressure using rotator evaporator (Rotavapor ® R-210) (10-15 rpm at 50- 55°C). The extraction was done in Pharmacognosy laboratory, Department of pharmacology, College of Medicine, University of Lagos, Lagos Nigeria.

***Acute Oral Toxicity Study of Seneciobiafrae Extract:*** The acute oral toxicity study for *Seneciobiafrae* extract was conducted using the Organization for Economic Cooperation and Development (OECD). Guidance Document on Humane end points that should reduce the overall suffering of animals used in this type of toxicity test. The test used was the limit dose test of the up and down procedure. Briefly, 5 animals were weighed and individually identified. The first animal was given the test dose – *Seneciobiafrae *extract 2000 mg per kg body weight. The second and third animals were concurrently dosed and the fourth and fifth animals sequentially dosed. The results were evaluated as follows (S = Survival, X = death). The animals were observed individually at least once during the first 30 minutes after dosing, periodically during the first 24 hours (with special attention given during the first 4 hours), and daily thereafter for a total period of 14 days. All observations were systematically recorded with individual records maintained for each animal.

***Phytochemical screening:*** Qualitative and Quantitative phytochemical analysis of the aqueous leaf extract of *Seneciobiafrae* was done in accordance with Soni and Sosa (22). While modifications on the report by Grindberg and Williams (23), on high performance liquid chromatography was adopted to quantify the vitamins and determination of minerals content: The sodium, calcium, Potassium, iron, zinc and phosphorus were determined using the method described by Akubugwo* et al.* (24).

***Experimental Animals and Treatment:*** Twenty-four adult male sprague dawley Rats weighing 180-200g, aged 10-12 weeks, were obtained from the Animal House of Department of Anatomy, Ladoke Akintola University of Technology, Ogbomoso. The animals were housed in cages at room temperature, under controlled environmental conditions, 12/12 hour light/dark cycle, and given water and food *ad libitum *and allowed to acclimatize for a period of two week before the commencement of the experiment. The animals were randomly grouped into four groups consisted of 6 rats each. Group A received 2mls of distilled water; group B given 500 mg/kg of *Senecio biafrae* extract; group C treated with 500 mg/kg of *Senecio biafrae* extract and 0.5 mg/kg zinc sulfate; and group D fed with 500 mg/kg of *Senecio biafrae* extract and1 mg/kg of Zn. The zinc sulfate used in this study is a zinc sulfate heptahydrate (ZnSO4.7H20) from Merck (Darmstadt Germany). The dosage of zinc sulfate used in this study refers to the work (25). All of the test materials were administered once daily via gastric gavage for 28 days. All experimental procedures followed the recommendations provided in the “Guide for the Care and Use of Laboratory Animals” prepared by the National Academy of Sciences and Published by the National Institute of Health (26).

***Blood Sampling and Organs Extraction*** *: *Twenty-four hours after the last administration, all the rats were sacrificed by cervical dislocation. Blood sample was collected by cardiac puncture and allowed to clot at room temperature for 45 minutes. Sera then separated by centrifugation at 2500 rpm for 15 minutes and kept frozen at -20°C for hormone assays. 

Testis and epididymis were taken by dissecting the peritoneal cavity at the posterior part of the abdomen. Any excess fat or connective tissue removed from the sampled organs, and then testis, epididymal, seminal vesicle, vas deference and prostate gland were weighed using analytical-density digital balance with readability of 0.0001 g. The testes were fixed in specimen bottles containing Bouin’s fluid for histological studies.

**Estimation of Haematological parameters: **Estimation of haematological parameters was done by method reported by Ibegbu *et al.* (27). Blood was collected by means of Cardiac puncture and blood cell count was done using an auto-analyzer. Red blood cell count (RBC), mean corpuscular hemoglobin (MCH), platelet count, white Blood cell count (WBC), packed cell volume were analyzed and differential white blood cell count was done.

**Testosterone assay: **Radioimmunoassay (RIA) for serum and testicular testosterone was carried out with a testosterone 125I RIA Kit (ICN, Biochemical, Immunotech, Marseille, France) according to the manufacturer’s protocol, as reported previously (28). Radioactivity was determined by gamma scintillation counting. All samples were run in duplicate in a single assay to avoid interassay variation.

***Sperm count and motility:*** Assessment of sperm count and motility was performed according to the method of Freund and Carol (29). The two caudaepididymis from each rat were placed in 2 ml of warmed (37°C) Earle’s buffer. Sperm count and motility were examined using Mallassez cells and the light microscope as described by Hoppe and Pitts (30).

***Testicular histology preparation: ***The histology of the testes was done by modification of method reported by Akang* et al.,* (31). The organs were harvested and fixed in Bouin‘s fluid for 24 h after which it was transferred to 70% alcohol for dehydration. The tissues were passed through 90% and absolute alcohol and xylene for different durations before they were transferred into two changes of molten paraffin wax for 1 hour each in an oven at 65◦C for infiltration. They were subsequently embedded and serial sections cut using rotary microtome at 5 microns. The tissues were picked up with albumenized slides and allowed to dry on hot plate for 2 min. The slides were dewaxed with xyleneand passed through absolute alcohol (2 changes); 70% alcohol, 50% alcohol and then to water for 5 min. The slides were then stained with haematoxylin and eosin. The slides were mounted in DPX. Photomicrographs were taken at a magnification of x100.

***Data presentation and statistical analysis:*** Data were expressed as mean ± standard error of mean. Statistical differences between the groups were evaluated by one way ANOVA, followed by Dunnets comparison test to compare between treated and control groups. p < 0.05 was considered significant. Statistical analyses of data were performed using GraphPad prism version 5.00 software.

## Results

***Acute oral Toxicity Studies: ***There were no deaths of rats dosed 3000 mg/kg body weight of the plants extract both within the short and long outcome of the limit dose test of Up and Down method (Table 1). The LD50 was calculated to be greater than 3000 mg/kg body weight /orally.

**Table 1:** This table shows results of acute toxicity test for Seneciobiafrae (up and down procedure) in rats

**Test serial number**	**Animal Identity**	**Dose of ** ***S. biafrae*** **mg/kg**	**Short term result ** **(48hrs)**	**Long term results ** **(14days)**
1	I	3000	S	S
2	LLT	3000	S	S
3	RLT	3000	S	S
4	TC	3000	S	S
5	LEP	3000	S	S
6	REP	3000	S	S

S = Survival; REP = Right ear pierced; LEP = Left ear pierced; TC = Tail cut; RLT= Right leg tagged; LLT=Left Leg tagged, I = Intact rat

***Phytochemical screening:*** Qualitative analysis of *Seneciobiafrae *aqueous crude leaves extract shows the presence of alkaloids, phlobatannins, flavonoids, tannins, terpenoids, cardiac glycoside, saponins, steroids, sodium, potassium, calcium and phosphorous (Table 2). After the quantitative analysis, total saponins, total phenol and total flavonoids had higher values compared to total taninins and the total alkaloids present. There were also high values of vitamins A, C, D and E (Table 3).

**Table 2:** Qualitative Phytochemical Analysis of Aqueous crude Extract of Seneciobiafrae

**S/N**	**Phytochemicals**	**Status**
1	Flavonoids	+
2	Tannins	+
3	Saponins	+
4	Steroids	+
5	Alkaloids	+
6	Cardiac glycosides	+
7	Terpenoids	+
8	Phlobatannins	+
9	Quinones	-
10	Coumerins	-
11	Sodium	+
12	Potassium	+
13	Calcium	+
14	Phosphorous	+
15	Zinc	-
16	Iron	-

+ Present, - not present

***Change in body weight and genital organs:*** There was no significant (p > 0.05) increase in the changes observed in the testis, epididymal, seminal vesicle, vas deference and prostate gland weight of animals across the group receiving the *Seneciobiafrae *aqueous crude leaves extract combine with Zn however there was significant (p < 0.05) increased in body weight of the animals across the group (Table 4).

**Table 3:** Quantitative phytochemical analysis of aqueous crude extract of Seneciobiafrae

**S/N**	**Phytochemicals**	**Quantity**
1	Vitamin A (mg/g)	4.03
2	Vitamin C (mg/g)	4.45
3	Vitamin D (mg/g)	3.34
4	Vitamin E (mg/g)	6.23
5	Total Tannins (%)	4.54
6	Total Saponins (%)	14.12
7	Total Flavonoids (%)	10.43
8	Total Phenols (%)	16.26
9	Total Alkaloids (%)	6.25

***Sperm parameters:*** Sperm parameters of rats administered *Seneciobiafrae *aqueous crude leaves extract with or without zinc for 28 days are described in table 5. All sperm parameters namely sperm concentration, sperm viability, sperm motility, and sperm morphology markedly indicate a positive effect of *Seneciobiafrae *aqueous crude leaves extract with and without zinc supplement (p < 0.05) on the reproductive function of male rats. Specific to sperm motility, the effect of Zn supplementation even increased with the dose administered.

***Testosterone production: ***Testis and plasma testosterone showed a significant difference between control and treated groups, and specifically between the group administered only *Seneciobiafrae *aqueous crude leaves extract and the group that received combination of *Seneciobiafrae *aqueous crude leaves extract and Zn (p < 0.05). 

This shows that the administration of a combination of *Seneciobiafrae *aqueous crude leaves extract together with zinc in rats markedly increased testis and serum testosterone levels (Table 6).

**Table 4:** Effects of Senecio biafrae combined with zinc on body weight and genital organs of adult male Sprague dawley rats

**Parameters **	**Group A ** **(control)**	**Group B** **(500 mg/kg of ** ***S. *** ***biafrae*** ** extract)**	**Group C** **(500 mg/kg of ** ***S. biafrae*** **extract + 0.5mg/kg of zinc)**	**Group D** **(500 mg/kg of ** ***S. biafrae*** **extract + 1mg/kg of zinc)**
Initial body weight (g)	193.80 ± 1.62	192.20 ± 1.25	193.50 ± 1.54	192.80 ± 0.98
Final body weight (g)	204.50 ± 0.56	205.20 ± 0.60*	206.50 ± 0.76*	209.50 ± 0.80*
Weight gain (g)	10.70 ± 1.06	13.00 ± 0.65	13.00 ± 0.78	16.70 ± 0.81
Testis weight (g)	1.78 ± 0.30	1.84 ± 0.42	1.86 ± 0.21	1.96 ± 0.24
Epididymis weight (g)	0.16 ± 0.01	0.15 ± 0.02	0.15 ± 0.01	0.14 ± 0.01
Seminal vesicle (g)	0.59 ± 0.01	0.61 ± 0.01	0.83 ± 0.01	0.87 ± 0.01
Vas deference (g)	0.12 ± 0.01	0.13 ± 0.01	0.14 ± 0.01	0.15 ± 0.01
Prostate gland (g)	0.35 ± 0.03	0.37 ± 0.02	0.38 ± 0.01	0.39 ± 0.04

Values are expressed as mean ± SEM for n = 6;

* p < 0.05, significantly dissimilar from control

Effects of SB extract + Zn on hematology parameters of male Sprague dawley rats are summarized in below (Table 7).

**Figure 1:**
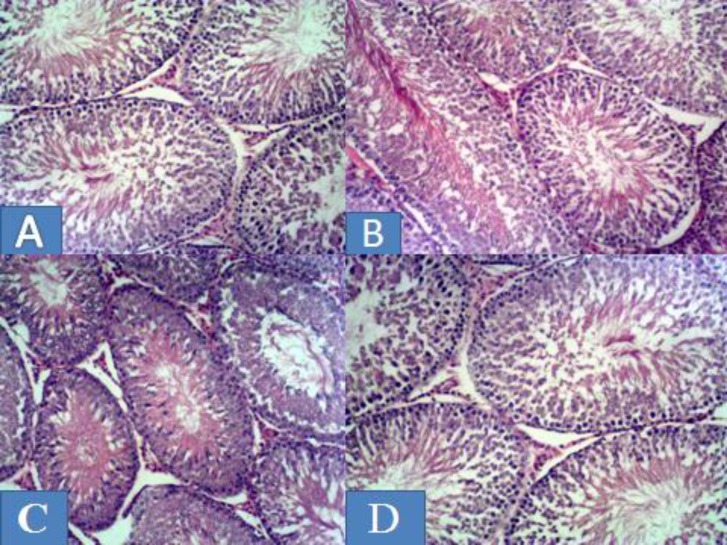
Photomicrographs of testis (×100) of Group A, the control, after 28days of administration, showing normal cellularity in germinal epithelium, lumen filled with sperm cells and interstitial cells of Leydig in the interstitium and normal seminiferous tubules with normal spermatogenesis; Group B, received of 500 mg/kgbwS. biafrae extract, revealed normal spermatogenesis and a better association of spermatogenic cells; Group C, administered with 500 mg/kgbw of S. biafrae extract and 0.5 mg/kgbw of Zn, also showed a normal spermatogenesis, better association and higher density of spermatogenic cells, Group D received 500 mg/kgbw of S. biafrae extract and 1mg/kg of Zn, showed normal spermatogenesis, very good association, spermatogenic cells denser and lumen contains full mature spermatozoa

***Testicular histology:*** Section of the testis of animals after 28 days of administration Group A, the control had a normal cellularity in germinal epithelium, lumen filled with sperm cells and interstitial cells of Leydig in the interstitium and normal seminiferous tubules with normal spermatogenesis; Group B, received of 500 mg/kgbw *Senecio biafrae* extract revealed normal spermatogenesis and a better association of spermatogenic cells; Group C, administered with 500 mg/kgbw of *Senecio biafrae *extract and 0.5 mg/kgbw of Zn, also showed a normal spermatogenesis, better association and higher density of spermatogenic cells, Group D received 500 mg/kgbw of *Seneciobiafrae *extract and 1mg/kg of zinc, showed normal spermatogenesis, very good association, spermatogenic cells denser and lumen contains full mature spermatozoa (Figure 1).

## Discussion

Present study was design to investigate role of aqueous crude leaf extract of *Seneci obiafrae *combined with Zn on testicular function of adult male sprague dawley rats. Testicular function is partly assessed by analysis of spermatic parameters including sperm count, motility, viability and morphology (32, 33). Measurements of these parameters in the spermatozoa give an indication of the quality and functionality of the sperm. As normal sperm motility and count are vital for male fecundity (32), increase in the number and motility of sperms by *Senecio biafrae *extract combined with Zn in our study indicates that the plant and Zn supplement could improve normal testicular function. Also, the percentages of viable sperms (i.e., spermatozoa with intact cell membrane) and morphologically abnormal sperms are critical indicators of testicular function (34). This result thus indicates that *Senecio biafrae* extract combined with zinc has an effect on the mitochondria found in the body of the spermatozoon where energy is been synthesis in the form of adenosine triphosphate, that increases the sperm motility (35).

**Table 5:** Effects of Senecio biafrae extract combined with zinc on sperm parameters of male Sprague dawley rats

**Parameters **	**Group A ** **(control)**	**Group B** **(500 mg/kbw of ** ***Seneciobiafrae *** **extract)**	**Group C** **(500 mg/kg of ** ***Seneciobiafrae *** **extract ** **+ 0.5mg/kg of zinc)**	**Group D** **(500 mg/kg of ** ***Seneciobiafrae *** **extract ** **+ 1mg/kg of zinc)**
Sperm concentration (x10^6^/ml)	25.50 ± 0.58	33.32 ± 1.48*	55.42 ± 1.37*	53.08 ± 0.95*
Sperm viability (%)	71.71 ± 0.82	78.78 ± 1.23*	91.35 ± 0.78*	90.04 ± 0.73*
Sperm motility (%)	69.26 ± 0.80	73.47 ± 1.09	78.24 ± 1.17*	83.20 ± 0.80*
Sperm abnomal (%)	10.17 ± 0.16	7.12 ± 0.11	5.15 ± 0.18*	3.79 ± 0.24*

Values are expressed as mean ± SEM for n = 6;

* p < 0.05, significantly dissimilar from control

**Table 6:** Effects of Senecio biafrae extract combined with zinc on testosterone production of male Sprague dawley rats

**Groups **	**Testosterone**	
	**Testis (ng/g)**	**Plasma (ng/ml)**
Group A (control)	3.97 ± 0.35	2.25 ± 0.20
Group B (500 mg/kb of *S. biafrae* extract)	5.18 ± 0.38*	2.98 ± 0.70
Group C (500 mg/kg of *S. biafrae* extract + 0.5mg/kg of zinc)	6.20 ± 0.42*	3.54 ± 0.31*
Group D (500 mg/kg of *S. biafrae* extract + 1mg/kg of zinc)	6.78 ± 0.38*	3.95 ± 0.30*

Values are expressed as mean ± SEM for n = 6;

* p < 0.05, significantly dissimilar from control

The testes have a dual function: spermatogenesis and steroid genesis. However, some conditions can interfere with spermatogenesis and reduce sperm quality and production. Various factors such as medication, chemotherapy, toxins, polluted air, lack of nutrients and vitamins can adversely affect spermatogenesis and sperm production (36). Similarly, in a natural or normal spermatogenesis, apoptosis can be taken place. Normal spermatogenesis is set appropriately and the balance between cell proliferation continuously and apoptosis (37).

Current findings showed that co-administration of *Senecio biafrae* and Zn, enhance spermatogenesis in rats with normal reproductive function. It can be assumed that the high effectiveness of *Senecio biafrae* on spermatogenesis along with Zn in this study was due to such an agent allegedly works through hypothalamus-pituitary-gonad axis. In this present study we discovered a significant increase in epididymal sperm concentration in the treated group (Table 5). The observed increased was due to the importance of *Senecio biafrae* as a potent antioxidant and freeradical scavenger (38). Therefore, support the report of Saalu* et al*., (39) that potent antioxidant ameliorate the increased free radicals generated by the natural and experimental stress, thereby increasing the spermatogenic activity by increasing the synthesis of testosterone from the interstitial cells of Leydig (39). Generally, there was significant increase in motility of sperm cells in all the treated groups as compared to the control group, also there was statistically significant increase in the number of progressive motile sperm cells in all the treated group. The abnormal progressive motile sperm cells number significantly decreased in group C and D but statistically significant in group B compared to the control group. 

Concerning the hormone level, our investigation also demonstrated that oral consumption of *Seneciobiafrae *extract combined with Zn increased the testosterone concentrations in plasma and testes therefore enhance spermatogenesis and steroid genesis (Table 6). Zn as been reported to play a key role in spermatogenesis (40). Zn in the Leydig cells, type-B spermatogonia and spermatids is essential for the production and secretion of testosterone from the Leydig cells (41). Together with follicle stimulating hormone, Zn is suggested as the key regulator of spermatogenesis (42). Conversely, the deficiency of Zn reduced function of the luteinizing hormone receptors, damages the Leydig cells and decreased synthesis of steroid (43). 

**Table 7:** Effects of Senecio biafrae extract combined with zinc on Hematology parameter of male Sprague dawley rats

**Parameters **	**Groups**			
	**Group A (control)**	**Group B(500 ** **mg/kgbw of ** ***S. biafrae*** **extract)**	**Group C(500 mg/kgbw ** **of ** ***S. biafrae*** ** extract + ** **0.5mg/kg of zinc)**	**Group D(500 mg/kgbw of ** ***S. biafrae*** ** extract + ** **1mg/kg of zinc)**
PCV(1/L)	0.50 ± 0.02	0.59 ± 0.02*	0.63 ± 0.02*	0.70 ± 0.03*
Hb(g/1)	15.28 ± 0.28	16.17 ± 0.26	16.73 ± 0.32*	16.88 ± 0.46*
NEUTROPHIL	23.20 ± 0.19	22.71 ± 0.30	22.25 ± 0.30	21.15 ± 0.33*
LYMPHOCYT	80.43 ± 0.63	81.97 ± 0.54	82.96 ± 0.56*	83.63 ± 0.50*
EOSINOPHIL	1.80 ± 0.10	2.33 ± 0.11*	2.58 ± 0.14*	3.17 ± 0.07*

Values are expressed as mean ± SEM for n = 6;

* p < 0.05, significantly dissimilar from control; Packed cell volume (PCV), Hemoglobin concentration (Hb).

Zn is required for DNA condensation and meiosis because it quite high in the developing spermatocytes and facilitates DNA packaging in spermatids (44, 45).

Phytochemical study of the *Senecio biafrae *revealed abundances of flavonoids, Saponins and Phenols. Flavonoids have been reported to posses potent inhibitory effect on enzymes involved in the production of the chemical mediators of inflammation and metabolism of arachidonic acid (46). Foods and fruits rich in flavonoids and other phenolic compounds have been associated with decreased risk of developing inflammatory and other related diseases (47) thus suggesting that the flavonoids in *Senecio biafrae *might be part of the anti-inflammatory constituents in the plant. From our observation, when *Senecio biafrae* aqueous leaves extract was administered combined with zinc; protect and improve normal functions of testis. This protective nature of *Senecio biafrae* is improved by some of its phytochemical constituents: the presence of ascorbic and folic acid which is known for its protection on cell membranes and its scavenging effects on free radicals (48, 49). In our finding we discovered presences of vitamin E in *Senecio biafrae *and in clinical trials, vitamin E supplementation has been found to increase fertilization rates possibly by enhancing membrane integrity, reducing oxidative damage and lipid peroxidation potential (50, 51).

The findings from this study have shown that *Senecio biafrae* is rich in antioxidant constituents such as flavonoids, saponins, vitamin E, vitamin C, vitamin D and vitamin A. This is in concordance with reports of Kayode and Kayode (52). We therefore deduced that these rich antioxidant constituent of *Senecio biafrae* could boosted the testicular non-enzymatic and enzymatic antioxidants to effectively scavenge free radicals and prevent lipid peroxidation. The consequence is hereby marked improvement in sperm count and sperm motility of the experimental group administered *Senecio biafrae *(Table 5). This finding is in concordance with the reports by (48, 53). Furthermore, vitamin E, a chain-breaking, non-enzymatic antioxidant also found in *Senecio biafrae* inhibits lipid peroxidation in membranes by scavenging peroxyl (RO•) and alkoxyl (ROO•) radicals (54). The ability of vitamin E to maintain a steady state rate of peroxyl radical reduction in the plasma membrane depends on the recycling of vitamin E by external reducing agents such as ascorbate (present in *Senecio biafrae*) or thiols Saleh and Agarwal (54). The improved sperm parameters are also attributed to the amino acid content of *Senecio biafrae *(55). Amino acids such as alanine, glycine, cystine and arginine which are present in *Senecio biafrae* have been reported to preserve sperm cells and improve their motility (56). Similarly present investigation clearly demonstrated that the treatment of rats with *Senecio biafrae *combined with Zn positively impact upon sperm quality parameters, as manifested by an increased in sperm motility and sperm count. The improvement of sperm quality may be due to the antioxidant components of *Senecio biafrae*, such as α – tochopherol (vitamin E), ascorbic acid (vitamin C) and selenium that improve testicular functions and sperm quality (57, 58). Vitamin C is a well-known antioxidant that is present in the test is protecting it from oxidative damage (59). Thus, it has been recently reported that the decreased in the testicular level of vitamin C are correlated with methylparathion-mediated effects on sperm quality and count in rats (60).

Treatment of rats with aqueous extract of *Senecio biafrae *combined with Zn in this study shown that co-administrion of *Senecio biafrae *and Zn has a potential to increase mean bodyweights of rats, the changes in the mean bodyweight was statistically significant, when compared to the control group (p < 0.05). There was slight increase in weight of epididymis, seminal vesicles, vas deference and prostate gland in the test groups when compared with the control group, though it was not statistically significant despite the increase in cellular activity in the testes. This is in line as reported by Shittu *et al*. (61) that increased cellular activities are key factor to be considered in the evaluation of organ weights.

There is no any observable lesion in the histology of the testes in the entire extract groups when compared with the control. Plants containing flavonoids are effective in prevention of lesion, mainly because of their antioxidant properties (62, 63). However, in all the test groups, there was observed increase in spermatogenic activities towards the lumen of the seminiferous tubule. This increase cellular activity was from the basement membrane up to the lumen of the seminiferous tubules of the testes. This was evidenced by the reduced number of primary spermatogonia cells. This is an indication that they might have differentiated to next level of spermatogenic cells. This was mainly due to the presence of potent antioxidant like favonoids that sacavange free radicals and increase testosterone formation by the interstitial cells of Leydig (39).

## Conclusion

Combination of aqueous extract of *Seneciobiafrae *and Zn therefore improved testicular function including steroidogenesis and spermatogenesis in rats, addition of zinc into *Seneciobiafrae *can be used as potential fertility herbs in male.
